# Anti-viral action against type 1 diabetes autoimmunity: The GPPAD-AVAnT1A study protocol

**DOI:** 10.1016/j.conctc.2025.101434

**Published:** 2025-01-20

**Authors:** Sandra Hummel, Alexandra Käßl, Stefanie Arnolds, Peter Achenbach, Reinhard Berner, Kristina Casteels, Heikki Hyöty, Olga Kordonouri, Helena Elding Larsson, Markus Lundgren, M. Loredana Marcovecchio, Catherine Owen, Markus Pfirrmann, Steve Robson, Agnieszka Szadkowska, Agnieszka Szypowska, Timothy Tree, Andreas Weiss, Anette-Gabriele Ziegler, Ezio Bonifacio, GPPAD Study Group

**Affiliations:** a Institute of Diabetes Research, Helmholtz Munich, German Research Center for Environmental Health, Munich, Germany; b Technical University Munich, School of Medicine and Health, Forschergruppe Diabetes at Klinikum rechts der Isar, Munich, Germany; c Department of Pediatrics, University Hospital Carl Gustav Carus, Technische Universität Dresden, Dresden, Germany; d Department of Pedriatrics, University Hospitals Leuven, Leuven, Belgium; e Department of Development and Regeneration, KU Leuven, Leuven, Belgium; f Faculty of Medicine and Health Technology, Tampere University, Tampere, Finland; g Kinder- und Jugendkrankenhaus AUF DER BULT, Hannover, Germany; h Department of Paediatrics, Skane University Hospital, Malmö, Sweden; i Department of Clinical Sciences Malmö, Lund University, Lund, Sweden; j Department of Pediatrics, Kristianstad Hospital, Kristianstad, Sweden; k University of Cambridge, Department of Paediatrics, and Cambridge University Hospitals NHS Foundation Trust, Cambridge, UK; l General Paediatrics, The Great North Children's Hospital, Royal Victoria Infirmary, Newcastle upon Tyne, UK; m Institut für Medizinische Informationsverarbeitung, Biometrie und Epidemiologie, Ludwig-Maximilians-Universität München, Munich, Germany; n Department of Paediatrics, Diabetology, Endocrinology and Nephrology, Medical University of Lodz, Lodz, Poland; o Department of Paediatric Diabetology and Paediatrics, Medical University of Warsaw, Warsaw, Poland; p Department of Immunobiology, School of Immunology & Microbial Sciences, King's College London, UK; q Center for Regenerative Therapies Dresden (CRTD), Faculty of Medicine, Technische Universität Dresden, Dresden, Germany; r Paul Langerhans Institute Dresden of the Helmholtz Munich at University Hospital Carl Gustav Carus and Faculty of Medicine, TU Dresden, Germany

**Keywords:** Islet autoimmunity, Type 1 diabetes, vaccination, COVID-19, Infection

## Abstract

Viral infections in the first year of life are associated with islet autoimmunity and type 1 diabetes risk. The Anti-Viral Action against Type 1 Diabetes Autoimmunity (AVAnT1A)- study is a clinical phase IV investigator initiated, randomised, controlled, multicentre, primary prevention trial conducted to determine whether vaccination against COVID-19 from 6 months of age reduces the cumulative incidence of islet autoantibodies or type 1 diabetes in children with elevated genetic risk. Additionally, it investigates the role of viral infections in the etiology of islet autoimmunity by intense surveillance within the first two years of life. Infants aged 3.00–4.00 months from Germany, Belgium, UK and Sweden are eligible if they have a >10 % expected risk to develop islet autoantibodies by age 6 years as determined by HLA DR/DQ genotype, polygenic risk score and family history of type 1 diabetes. A total of 2252 eligible children are randomized 1:1 to COVID-19 vaccine (Comirnaty® 3 μg Omicron XBB.1.5 or future new variants) or placebo (0.9 % Sodium Chloride) administered three times. Children are followed until the minimum age of 2.5 years and maximum age of 6 years. The intervention is accompanied by analyses of immune and metabolic parameters to determine changes induced by viral infections and to investigate mechanisms by which viral infection may lead to islet autoimmunity. The Sponsor is the Klinikum rechts der Isar, Technical University Munich. The study was approved by Clinical Trials Information System (CTIS, EU Trial number: 2023-507348-35-00) and by Integrated Research Application System (IRAS, IRAS-ID: 1009668).

## 1 Introduction

Type 1 diabetes results from an immune-mediated destruction of the pancreatic islet beta cells leading to insulin deficiency. This process is clinically silent and can be identified by circulating autoantibodies to islet antigens (GAD65 (GADA) [1], IA-2 (IA-2A) [2,3] insulin (IAA) [4] and ZnT8 (ZnT8A) [5]). Islet autoantibody development has a peak incidence period between age 9 months and 3 years demonstrated in German [6], Finnish [7] and in multicenter studies [8]. In an analysis of over 13,000 prospectively followed children, 80 % of those who developed type 1 diabetes before the age of 20 years already developed islet autoantibodies before the age of 5 years [9]. Almost all children who develop the stage of multiple islet autoantibodies progress to clinical diabetes [9]. The earlier the process of islet autoimmunity is initiated, the more rapid is the progression to type 1 diabetes. Therefore, intervention therapy as a primary prevention strategy must be started early in life and target key early childhood events that may modify genetic susceptibility for type 1 diabetes.

Previous epidemiological and genetic data associated viral infections with type 1 diabetes. Infections in the first year of life increase the risk of islet autoimmunity and type 1 diabetes [[10], [11], [12]]. A large study of claims data showed that children with two or more infections by 6 months of age were more than twice as likely to develop type 1 diabetes by 8 years of age [13]. Furthermore, children who develop islet autoimmunity experience their first viral infection earlier than children who did not develop islet autoimmunity [10]. An increased frequency of viral infections in the time window before the first appearance of islet autoantibodies is also reported [11,12]. These data suggest that early childhood infections and infections shortly before the onset of autoimmunity may promote autoreactivity towards islet cells. Furthermore, multiple virus response genes are linked to the risk of developing islet autoimmunity [14]. Repeated attempts to identify which viruses are responsible for the increased risk of autoimmunity and diabetes suggest enteroviruses, particularly coxsackie B virus, as potential candidates.

Recent studies also suggest a contribution of the severe acute respiratory syndrome coronavirus 2 (SARS-CoV-2) to the development of type 1 diabetes. In addition to reports of cases with type 1 diabetes that occur after COVID-19 [15], and despite a general reduction of infection rates in young children during the pandemic [16], an increased incidence rate of childhood diabetes since the start of the pandemic is observed in most studies [17,18]. In a study of over 1.1 million statutorily insured children born between 2010 and 2018 in Bavaria, Germany, a temporal association between COVID-19 and the development of type 1 diabetes was observed [19]. In children with multiple islet autoantibodies from the Fr1da-project, an almost two-fold increased rate of progression to clinical type 1 diabetes after COVID-19 infection was observed providing the first evidence that COVID-19 infection accelerates the disease process [20]. Consistent with these findings, the POInT study, which longitudinally followed 1050 newborns with increased genetic risk for developing multiple islet autoantibodies from 2018 to 2024 [21], showed a temporal association between COVID-19 and the development of islet autoantibodies in early childhood [22]. In particular, a COVID-19 infection before 18 months of age increased the risk of islet autoantibodies more than 5-fold in these children. COVID-19 infections in teenagers were not associated with an increased risk to develop islet autoantibodies, consistent with the notion that infections earlier in life are critical [23].

Children with increased risk for islet autoimmunity and type 1 diabetes also have an increased risk of developing celiac disease-associated transglutaminase autoantibodies [24,25]. There is no clear evidence that COVID-19 infection leads to an increase in celiac disease incidence, though more research on this topic with longer-term follow-up is necessary to make this assessment [26]. Similar to islet beta cells, intestinal enterocytes express the ACE2 receptor, and a theoretical risk of an increased incidence of celiac disease as a consequence of COVID-19 has been hypothesized.

Taken together, these findings suggest that viral infections modify the risk of autoimmunity in early life in children who are genetically susceptible and that vaccination against key early childhood infections offers a strategic approach for the primary prevention of autoimmune diseases such as type 1 diabetes. The Global Platform for the Prevention of Autoimmune Diabetes (GPPAD) - Anti-Viral Action against Type 1 Diabetes Autoimmunity (AVAnT1A)- study is a clinical phase IV investigator initiated, randomised, controlled, multicentre, multinational, primary prevention trial (CT) conducted to determine whether vaccination against COVID-19 from 6 months of age reduces the cumulative incidence of islet autoantibodies or type 1 diabetes in children with elevated genetic risk.

## 2 Objectives

### 2.1 Primary objective

The AVAnT1A-CT is conducted to determine whether vaccination of children with elevated genetic risk for type 1 diabetes against COVID-19 from age 6 months will reduce the cumulative incidence of islet autoantibodies or type 1 diabetes in childhood.

### 2.2 Secondary objectives

The secondary objectives include determining whether vaccination against COVID-19 similarly reduces the cumulative incidence of multiple islet autoantibodies, of type 1 diabetes and of celiac disease-associated transglutaminase autoantibodies in childhood.

### 2.3 Exploratory objectives

Exploratory objectives include examinations to determine whether COVID-19 vaccination influences the frequency and features of islet autoreactive T cells, the effects of COVID-19 vaccination on glucose metabolism and pancreatic function, and how immune parameters alter in response to COVID-19 vaccination, whether COVID-19 infection and/or the severity of infection are associated with the development of islet autoimmunity, whether maternally acquired SARS-CoV-2 or other anti-viral antibodies reduce islet autoantibody risk.

Furthermore, the rate of specific viral infections (SARS CoV-2, Enteroviruses, Rotavirus, Human Coronaviruses-NL63, -229E, -OC43, and -HUK1, influenza A, Rhinovirus, Adenovirus, Bocavirus, Norovirus, Astrovirus) over the first 2 years of life will be investigated as well as a possible temporal association of such viral infections with the development of islet autoimmunity.

## 3 Outcomes

### 3.1 Primary efficacy outcome

The primary efficacy outcome is the elapsed time from random treatment assignment to the development of persistent confirmed islet autoantibodies or type 1 diabetes. For subjects who will develop persistent confirmed islet autoantibodies, the elapsed time will be from the random treatment assignment to the first confirmed autoantibody positive sample used in defining the persistent confirmed islet autoantibody positive status. A diagnosis of type 1 diabetes will be realized by Oral Glucose Tolerance Test (OGTT) criteria for diabetes or clinical criteria for diabetes according to the American Diabetes Association (ADA) [27] definition.

Islet autoantibody status is based on the measurement of islet autoantibodies against IAA, GADA, IA-2A, and ZnT8A tested in the GPPAD central autoantibody laboratory (Helmholtz Munich). Confirmed islet autoantibody is defined as sample positive for one specific antibody in both a screening and confirmatory assay that has a different format to the screening assay. The status persistent confirmed islet autoantibody-positive is defined as confirmed IAA, confirmed GADA, confirmed IA-2A, or confirmed ZnT8A in two consecutive samples. Persistence of at least one confirmed islet autoantibody until the last follow-up sample is required for an outcome of persistent confirmed islet autoantibody.

For children who test positive for any of the four islet autoantibodies in the first sample collected and do not have a negative sample prior to this sample, the autoantibody status will be classified as maternally derived islet autoantibodies if the autoantibodies show a continuous decline in titer and/or become negative in subsequent sample(s) collected before the age of 2.5 years. Islet autoantibodies that will be considered maternally derived are NOT positive for the primary outcome.

### 3.2 Secondary outcomes

Secondary outcomes are the elapsed time from random treatment assignment to the development of persistent confirmed multiple islet autoantibodies; the development of type 1 diabetes; the development of persistent confirmed transglutaminase autoantibodies. Persistent confirmed multiple islet autoantibodies is defined as confirmed IAA, confirmed GADA, confirmed IA-2A, or confirmed ZnT8A in two consecutive samples, AND a confirmed second antibody from these four antibodies in one sample. Persistent confirmed transglutaminase autoantibodies are defined as sample positive for transglutaminase autoantibodies in both a screening and confirmatory assay that has a different format to the screening assay. The status persistent confirmed transglutaminase autoantibodies positive is defined as confirmed transglutaminase autoantibodies in two consecutive samples.

### 3.3 Exploratory measures

• Anti-viral antibodies in blood measured by multiplexed anti-viral antibody panel (including SARS CoV-2, Enteroviruses, Rotavirus, Human Coronaviruses-NL63, -229E, -OC43, and -HUK1, influenza A, Rhinovirus, Adenovirus, Bocavirus, Norovirus, Astrovirus)
• Saliva virus qPCR panel (SARS CoV-2, Enterovirus, Human Coronaviruses-NL63, -229E, -OC43, and -HUK1, influenza A, Rhinovirus, Adenovirus, Bocavirus)
• Stool virus qPCR panel (Enterovirus, Rotavirus, Adenovirus, Rhinovirus, Norovirus, Astrovirus)
• Islet autoantigen specific T cell response and single cell RNAseq of the responsive cells
• Whole blood multiparameter flow counts
• inflammatory markers
• in vitro immune cell response to viral and autoantigens and general stimuli
• Whole blood transcriptomics
• Whole blood DNA methylation
• Single cell multiomics
• Pre-prandial and post-prandial blood glucose
• C-peptide, proinsulin, insulin
• HbA1c
• Serum Metabolomics
• Plasma vitamin D3 concentration
• OGTT in islet autoantibody positive children
• Weight-for-length, Body Mass Index

## 4 Hypotheses

The primary hypothesis to be tested in the AVAnT1A-CT is that vaccination against COVID-19 is superior to placebo in preventing the development of islet autoantibodies or type 1 diabetes in childhood. Additional secondary and exploratory hypotheses include COVID-19 vaccination prevents the development of celiac disease-associated transglutaminase autoantibodies in childhood, infections with specific viruses influence the risk for beta cell autoimmunity and infection influences immune trajectories in early childhood.

## 5 Trial design

AVAnT1A-CT is a randomised, multicentre, multinational, primary prevention trial for children at increased risk of type 1 diabetes starting from age 3.00–4.00 months. Screening for increased genetic risk is performed in the context of newborn screening with separate study protocols and consents as previously described [28]. The trial Sponsor is the Klinikum rechts der Isar, Technical University Munich, Germany. Participating sites and countries are Belgium (Leuven), Germany (Dresden, Hanover, Munich), Sweden (Malmö/Lund), and UK (Cambridge, Newcastle). Enrolment commenced in May 2024 (First Patient First Visit).

The study duration for individual children will depend upon when they are enrolled into the study. The minimum duration is from age 3.00–4.00 months until age 2.5 years. This will apply to the last child enrolled into the study. The first child enrolled into the study will have a study duration from age 3.00–4.00 months until age 6 years. The period to enrol all 2252 subjects is expected to take 39 months (expected last patient last visit in October 2029). From age 6 months, children will be randomised 1:1 to COVID-19 vaccine or placebo and will be administered three vaccinations. The study is conducted with the vaccine Comirnaty® 3 μg Omicron XBB.1.5 (until October 2024) and Comirnaty® 3 μg JN.1 from November 2024, and future COVID-19 variant vaccines as they become available. Saline (0.9 % sodium chloride solution for injection) is used as placebo. Vaccination against COVID-19 among children aged 6 months to 4 years remains low across all participating countries, with an estimated utilization rate of less than 0.1 %. Therefore, children receiving the placebo effectively receive the current standard of care. In the case that regulatory or public health guidance change to recommend routine COVID-19 vaccination for children under age 2 years, the feasibility as well as ethical considerations of a placebo-controlled study will be discussed with regulatory authorities.

Children in both Comirnaty (Comirnaty® 3 μg Omicron XBB.1.5 or JN.1) and placebo arms are followed by a blinded follow-up (FU) period. Procedures are summarized in Appendix A. The Trial Coordination Centre (GPPAD CC) will be responsible for the coordination and communication between the clinical study sites of AVAnT1A and controls the collection, analysis and storage of clinical data. It will also be responsible for monitoring the regulatory activities, the activities of the clinical research organisation and the central laboratories. It is located at the Institute of Diabetes Research at Helmholtz Zentrum München. An independent Data Safety Monitoring Board (DSMB) is commissioned to continually assess the conduct of study. Periodic review of clinical data will also be conducted by a medical monitor.

## 6 Participant identification

### 6.1 Trial participants

Screening for genetic risk is organized outside of AVAnT1A-CT. Children are tested for genetic risk of type 1 diabetes based on risk scores derived from SNPs that define HLA DR3, HLA DR4, HLA DQ8 and HLA DQ7 alleles as well as SNPs from the HLA region, including HLA class II protective alleles and non-HLA type 1 diabetes susceptibility genes. Children with a predicted risk of >10 % to develop islet autoimmunity by age 6 years and who fulfil the inclusion criteria as stated below will be asked to participate in AVAnT1A-CT.

### 6.2 Inclusion criteria

Each potential subject must satisfy all of the following criteria in order to permit enrolment in the study:

1. Age between 3.00 and 4.00 months at the time of enrolment.
2. A high genetic risk (>10 %) to develop islet autoantibodies by age 6 years:
a) For children without a first-degree family history of type 1 diabetes, high genetic risk is defined as a DR3/DR4-DQ8, DR4-DQ8/DR4-DQ8 or DR4-DQ8/DR4-DQ7 and an rs6901541 C/T genotype and an elevated sex-specific genetic risk score that is at the 98.75th centile, in other words identifies around 1.25 % of newborns without a first-degree family history with type 1 diabetes.
b) For children with a first-degree family history of type 1 diabetes, high genetic risk is defined as having HLA DR4 and DQ8, none of the following protective alleles: DRB1∗1501, DQB1∗0503, DRB1∗1303, and a sex-specific genetic risk score >50th centile of the background population. These represent around 25 % of children with a first-degree family history of type 1 diabetes.
3. Written informed consent signed by the custodial parent(s).

### 6.3 Exclusion criteria

Any potential subject who meets any of the following criteria will be excluded from participating in the study:

1. Previous hypersensitivity to the excipients of the vaccine. These include:
  ((4-hydroxybutyl)azanediyl)bis(hexane-6,1-diyl)bis(2-hexyldecanoate) (ALC-0315)
  2-[(polyethylene glycol)-2000]-N,N-ditetradecylacetamide (ALC-0159)
  1,2-Distearoyl-sn-glycero-3-phosphocholine (DSPC)
  cholesterol
  trometamol
  trometamol hydrochloride
  sucrose
2. Any medical condition, concomitant disease or treatment that may interfere with the assessments or may jeopardize the participant's safe participation in the study. These include immune deficiencies, and conditions or treatments that lead to immune suppression.
3. Likely poor compliance due to expected change in residency.
4. Diagnosis of diabetes prior to recruitment or randomisation.
5. Current use of any other investigational drug.

## 7 Trial procedure

### 7.1 Informed consent

The AVAnT1A-CT is described to the custodial parent(s) of potential participants by qualified study personnel. The custodial parent(s) will have the opportunity to read the consent document and to discuss any questions concerning the consent or study participation. The families will be given enough time to consider whether or not to participate. The custodial parent(s) are asked to sign and date an informed consent form prior to or at the baseline visit. Date and signature of the study Investigator are also obtained on the consent form. A copy of the informed consent form is handed out to the families. The custodial parent(s) of the prospective participant are told that being in the study is voluntary and that the participant may withdraw from the study at any time, for any reason.

Patients were involved in prioritising the research question of type 1 diabetes prevention, but not in the study design. They will promote recruitment by disseminating information and attending press conferences. At the end of study, participating families will be informed about study results by webcast, letter and personal communication.

### 7.2 Study visit assessments

Clinical assessments, study procedures, and collection of biosamples is summarized in Appendix A. Clinical assessments include the collection of information on medical history, body weight and height measurements, physical examination, capillary or venous blood draw, saliva collection, vaccination as well as the assessment of Adverse Events (AEs) and Serious Adverse Events (SAEs).

Biosamples include all samples listed in exploratory outcomes. All study relevant subject data and laboratory results will be documented in corresponding electronic case report forms (eCRFs).

**E-diaries:** From baseline visit until age of 36 months parents will be asked to complete e-diaries fortnightly until age 36 months ± 14 days to collect information on infections – especially respiratory and gastrointestinal infections – fever, chronic diseases and breastfeeding. Information provided by the parents will be recorded in a central database and reviewed and discussed during the study visits.

**Psychological impact:** The psychological effect of study participation will be monitored by a questionnaire at visit 5 (age 12 months) and visit 9 (age 24 months). The questionnaire will preferably be completed by each of both parents or custodial parent(s). The questionnaire was previously used in the POInT study [29]. When a parent is identified with high levels of anxiety and/or distress (PHQ-D/A; diabetes-specific items), a structured concept of psychological care will be provided [29].

### 7.3 For participants who develop positive islet or transglutaminase autoantibodies

Custodial parent(s) are informed when a child develops persistent confirmed islet autoantibodies. The parents are asked to participate in an educational program informing about the diagnosis of islet autoantibody positivity. Contents of the education will be information what the diagnosis “islet autoantibodies” means, how to recognize clinical symptoms of type 1 diabetes and how to self-monitor blood glucose. Home monitoring of blood glucose will be recommended if a child has clinical symptoms of diabetes, or any infection or fever; home monitoring will also be recommended routinely every 2-months if a child is considered at risk for a rapid progression to diabetes (e.g. IA-2A positive, very high titers of antibodies, or impaired blood glucose values). Children who develop persistent confirmed islet autoantibodies will have OGTTs including assessments of C-peptide from age 3 years at each study visit. They will remain in the study until they develop clinical type 1 diabetes. Custodial parent(s) are also informed when a child has developed persistent confirmed transglutaminase autoantibodies. A consultation of the treating physician or specialist for celiac disease is recommended to them in this case. After completion of the AVANT1A-CT, children with islet or transglutaminase autoantibodies will be offered monitoring in the context of local early stage type 1 diabetes surveillance programs.

### 7.4 Known and potential risks and benefits

The potential benefits are regular monitoring and care of the child at high risk of type 1 diabetes and the prevention of diabetic ketoacidosis, symptoms, and hospitalization through early care. If islet autoimmunity is diagnosed, other disease-modifying therapies will possibly be available. Children randomized to vaccine are protected against severe COVID-19 infection and its early and late complication and are potentially protected from islet autoimmunity and diabetes.

Side effects of the Investigational Medicinal Product (IMP) will be minimal in comparison to the benefits of the approved IMP. Bood draws are frequent with the potential to lead to slight pain and bruising. The discomfort of frequent blood draws is minimized by the use of an anaesthetic cream. Follow-up is intense as compared to standard care and may be associated with excess family burden. To help families understand the reasons for the study and for the collection of blood, saliva and stool samples, substantial information materials that explain the study to families and children are provided. Further behavioural changes and psychological impact are monitored by questionnaires. Parents with a high level of anxiety or distress will be contacted and may be advised to consult a psychologist. Potential impedance to benefits of COVID-19 vaccination in children randomized to the placebo arm are mitigated by excluding children with conditions or treatment leading to immune deficiency and by informing families intending to vaccinate their children of the risk of receiving placebo and their options with respect to declining participation or withdrawal. Because of the current infrequent (<0.1 %) practice of vaccinating children below age 5 years in the participating countries the overall risk of impedance is considered low and considerably less than the benefit of providing vaccination to 50 % of the participants.

With the incorporated mitigation of risks, the overall benefits of participation are considered to outweigh the risk.

### 7.5 Retention

To make families feel part of the research team, a special family friendly retention concept will be in place. Participating families will benefit from special care and support; families will be reimbursed for travel expenses and children will receive small gifts during visits. The advantages of study participation will be clarified to parents, such as free monitoring of children's health status and the potential benefit of the intervention. Newsletters and reports on islet autoantibody tests as well as community building activities (homepage, Facebook groups) will also be used as a retention strategy. For further information refer to the GPPAD homepage: https://www.helmholtz-munich.de/en/idf/studies/avant1a-study.

## 8 Safety reporting

The investigators are responsible for the recording and reporting of AEs observed from randomisation (for UK sites: from consent) until 1 month after administration of the study medication. All AEs should be followed until event resolution or stabilisation or participant completion of the trial, whatever occurs first. The causality assessment of an Adverse Event Following Immunisation (AEFI) are outlined in the Safety Management Plan.

## 9 Statistics

All efficacy analyses will be performed on the modified intention-to-treat (mITT) population unless otherwise specified. The mITT population will include all randomised subjects who have received at least 1 dose of study drug. An analysis will also be performed on the per protocol population that will include all participants who complete the 3 vaccination visits. The overall difference between the groups in the cumulative incidence functions will be tested by the log-rank test at the two-sided significance level of 0.05. The hazard ratio of the two groups and its 95 % confidence interval will be determined by the Cox model. As a sensitivity analysis, the hazard ratio of the two groups will be assessed using the Cox regression when including site as covariate. The estimates of cumulative incidence and the log-rank test will adjust for periodic outcome assessment visits to assess islet autoantibody status. The secondary endpoints will be analysed using the same statistical methods as the primary endpoint.

### 9.1 Exploratory analyses

Exploratory analyses (inflammatory markers, immune cell populations, metabolomics, islet function markers, vitamin D3 concentrations, and virus exposure) may be assessed after completion and unblinding of the study and may be conducted later. Results of exploratory analyses will not be part of the clinical study report. A separate analysis plan on exploratory outcomes will be developed before these analyses are performed.

### 9.2 Study power and accrual target

The sample size calculation was based on the numbers needed to test the hypothesis that COVID-19 vaccination will reduce the incidence rate of islet autoantibodies in children with a high genetic risk to develop type 1 diabetes. The trial numbers were determined for 70 % power to detect a 26 % reduction in islet autoantibodies in the treated group at a two-tailed significance of 0.05. The expected infection rate until age 2.5 years used to determine the trial numbers will be 60 %. The trial numbers were based on a 2-fold increase in the risk for islet autoantibodies in infected versus non-infected children. Therefore, the risk by age 4 years is expected to be 8.75 % in uninfected children and 17.5 % in infected children. With a 60 % infection rate, the risk in the placebo group is expected to be 14 %. The study numbers are based on vaccination reducing the additional risk of 8.75 % in infected children by 70 %–2.625 %, resulting in a total risk of 11.375 % in infected children and an overall risk in the treatment arm of 10.3 %. The Hazard ratio with these assumptions is: Hazard in placebo group/hazard in vaccination group = 1.38397. With an accrual time of 3.25 years, an additional follow-up of 2 years and accounting for an assumed 15 % rate of loss to follow-up, a total of 2252 children would have to be included into the study.

## 10 Insurance

Study participants are insured with HDI-Gerling Industrie Versicherung AG.

Study participants are insured for all AEs resulting from study participation according to legal requirements (see also respective insurance policy). The insurance covers all direct or indirect damages that participants have experienced in the course of intervention with the study drug or by any study related test and examination procedures.

## 11 Approval status

The study was approved by the central submission authorities Clinical Trials Information System (CTIS) for European sites and by Integrated Research Application System (IRAS) for UK sites. In UK it was approved by the local authority Medicines and Healthcare products Regulatory Agency (MHRA) and by the ethics committee East of England - Cambridge South Research Ethics Committee as well as the Health Research Authority (HRA) and Health and Care Research Wales (HCRW). In addition it is registered at Clinical Trials.gov (NCT06452654) and responsible local authorities.

## 12 Dissemination

GPPAD is committed to data sharing in accordance with all applicable European and GPPAD Consortium Member State, Data Protection and Privacy Protection laws, rules and regulations.

## CRediT authorship contribution statement

**Sandra Hummel:** Writing – original draft, Conceptualization. **Alexandra Käßl:** Writing – original draft, Conceptualization. **Stefanie Arnolds:** Writing – original draft, Conceptualization. **Peter Achenbach:** Writing – review & editing, Conceptualization. **Reinhard Berner:** Writing – review & editing, Conceptualization. **Kristina Casteels:** Writing – review & editing, Conceptualization. **Heikki Hyöty:** Writing – review & editing, Conceptualization. **Olga Kordonouri:** Writing – review & editing, Conceptualization. **Helena Elding Larsson:** Writing – review & editing, Conceptualization. **Markus Lundgren:** Writing – review & editing, Conceptualization. **M. Loredana Marcovecchio:** Writing – review & editing, Conceptualization. **Catherine Owen:** Writing – review & editing, Conceptualization. **Markus Pfirrmann:** Writing – review & editing, Methodology, Conceptualization. **Steve Robson:** Writing – review & editing, Conceptualization. **Agnieszka Szadkowska:** Writing – review & editing, Conceptualization. **Agnieszka Szypowska:** Writing – review & editing, Conceptualization. **Timothy Tree:** Writing – review & editing, Conceptualization. **Andreas Weiss:** Writing – review & editing, Conceptualization. **Anette-Gabriele Ziegler:** Writing – original draft, Supervision, Funding acquisition, Conceptualization. **Ezio Bonifacio:** Writing – review & editing, Methodology, Conceptualization.

## Funding

The AVANT1A study is supported by the Leona M. and Harry B. 10.13039/100007028Helmsley Charitable Trust (Helmsley) grants #2302-06623 (GPPAD-02 study continuation 2), #2003–04286 (GPPAD coordinating center continuation), #2302–06624 (GPPAD-05 study: Antiviral Action against Type 1 diabetes Autoimmunity (AVAnT1A)), and funding from Helmholtz Munich, German Research Center for Environmental Health, Germany, the Bavarian 10.13039/501100004725Ministry of Economic Affairs, Energy, and Technology, Germany (grant: Prevention of Autoimmune Diabetes–10.13039/100031019Digital Lab), and EASD-10.13039/501100009708Novo Nordisk Foundation Diabetes Prize for Excellence NNF22SA0081044 to the German Clinical Center Munich. Funding from the Swedish Diabetes Foundation to the Swedish site, and the German Center for Diabetes Research (DZD e.V.) to the German Clinical Centers Munich and Dresden.

## Declaration of competing interest

The authors declare the following financial interests/personal relationships which may be considered as potential competing interests: Ezio Bonifacio reports financial support was provided by Leona M. and Harry B. Helmsley Charitable Trust. Reinhard Berner reports financial support was provided by Leona M. and Harry B. Helmsley Charitable Trust. Anette-Gabriele Ziegler reports a relationship with Sanofi that includes: board membership. Anette-Gabriele Ziegler reports a relationship with Provention Bio Inc that includes: board membership. Anette-Gabriele Ziegler reports a relationship with ITB-Med that includes: board membership. Anette-Gabriele Ziegler reports a relationship with Sanofi Aventis France that includes: board membership. M. Loredana Marcovecchio reports a relationship with Sanofi that includes: consulting or advisory. Anette-Gabriele Ziegler reports a relationship with Sanofi Germany that includes: speaking and lecture fees. Ezio Bonifacio reports a relationship with Sanofi that includes: speaking and lecture fees. Ezio Bonifacio reports a relationship with Sanofi that includes: travel reimbursement. Sandra Hummel reports a relationship with Sanofi that includes: travel reimbursement. Sandra Hummel reports a relationship with Novo Nordisk Pharma GmbH that includes: speaking and lecture fees. Reinhard Berner reports a relationship with Standing Committee on Vaccination (STIKO) that includes: board membership. Reinhard Berner reports a relationship with Standing Committee on Pandemic Respiratory Tract Infections at the RKI that includes: board membership. Reinhard Berner reports a relationship with Committee on Drug Safety and Drug Development for Children (KAKJ), Federal Insitute for Drugs and Medicinal Products (BfArM) that includes: board membership. If there are other authors, they declare that they have no known competing financial interests or personal relationships that could have appeared to influence the work reported in this paper.

## References

[1] Baekkeskov S., Aanstoot H.J., Christgau S., Reetz A., Solimena M., Cascalho M., Folli F., Richter-Olesen H., de Camilli P. (1990). Identification of the 64K autoantigen in insulin-dependent diabetes as the GABA-synthesizing enzyme glutamic acid decarboxylase. Nature.

[2] Rabin D.U., Pleasic S.M., Shapiro J.A., Yoo-Warren H., Oles J., Hicks J.M., Goldstein D.E., Rae P.M. (1994). Islet cell antigen 512 is a diabetes-specific islet autoantigen related to protein tyrosine phosphatases. J. Immunol..

[3] Lan M.S., Lu J., Goto Y., Notkins A.L. (1994). Molecular cloning and identification of a receptor-type protein tyrosine phosphatase, IA-2, from human insulinoma. DNA Cell Biol..

[4] Palmer J.P., Asplin C.M., Clemons P., Lyen K., Tatpati O., Raghu P.K., Paquette T.L. (1983). Insulin antibodies in insulin-dependent diabetics before insulin treatment. Science.

[5] Wenzlau J.M., Juhl K., Yu L., Moua O., Sarkar S.A., Gottlieb P., Rewers M., Eisenbarth G.S., Jensen J., Davidson H.W., Hutton J.C. (2007). The cation efflux transporter ZnT8 (Slc30A8) is a major autoantigen in human type 1 diabetes. Proc. Natl. Acad. Sci. U. S. A..

[6] Ziegler A.-G., Bonifacio E. (2012). Age-related islet autoantibody incidence in offspring of patients with type 1 diabetes. Diabetologia.

[7] Parikka V., Näntö-Salonen K., Saarinen M., Simell T., Ilonen J., Hyöty H., Veijola R., Knip M., Simell O. (2012). Early seroconversion and rapidly increasing autoantibody concentrations predict prepubertal manifestation of type 1 diabetes in children at genetic risk. Diabetologia.

[8] Krischer J.P., Lynch K.F., Schatz D.A., Ilonen J., Lernmark Å., Hagopian W.A., Rewers M.J., She J.-X., Simell O.G., Toppari J., Ziegler A.-G., Akolkar B., Bonifacio E. (2015). The 6 year incidence of diabetes-associated autoantibodies in genetically at-risk children: the TEDDY study. Diabetologia.

[9] Ziegler A.G., Rewers M., Simell O., Simell T., Lempainen J., Steck A., Winkler C., Ilonen J., Veijola R., Knip M., Bonifacio E., Eisenbarth G.S. (2013). Seroconversion to multiple islet autoantibodies and risk of progression to diabetes in children. JAMA.

[10] Mustonen N., Siljander H., Peet A., Tillmann V., Härkönen T., Ilonen J., Hyöty H., Knip M. (2018). Early childhood infections precede development of beta-cell autoimmunity and type 1 diabetes in children with HLA-conferred disease risk, Pediatr. Diabetes.

[11] Beyerlein A., Wehweck F., Ziegler A.-G., Pflueger M. (2013). Respiratory infections in early life and the development of islet autoimmunity in children at increased type 1 diabetes risk: evidence from the BABYDIET study. JAMA Pediatr..

[12] Lönnrot M., Lynch K.F., Elding Larsson H., Lernmark Å., Rewers M.J., Törn C., Burkhardt B.R., Briese T., Hagopian W.A., She J.-X., Simell O.G., Toppari J., Ziegler A.-G., Akolkar B., Krischer J.P., Hyöty H. (2017). Respiratory infections are temporally associated with initiation of type 1 diabetes autoimmunity: the TEDDY study. Diabetologia.

[13] Beyerlein A., Donnachie E., Jergens S., Ziegler A.-G. (2016). Infections in early life and development of type 1 diabetes. JAMA.

[14] Sioofy-Khojine A.-B., Richardson S.J., Locke J.M., Oikarinen S., Nurminen N., Laine A.-P., Downes K., Lempainen J., Todd J.A., Veijola R., Ilonen J., Knip M., Morgan N.G., Hyöty H., Peakman M., Eichmann M. (2022). Detection of enterovirus RNA in peripheral blood mononuclear cells correlates with the presence of the predisposing allele of the type 1 diabetes risk gene IFIH1 and with disease stage. Diabetologia.

[15] Hollstein T., Schulte D.M., Schulz J., Glück A., Ziegler A.G., Bonifacio E., Wendorff M., Franke A., Schreiber S., Bornstein S.R., Laudes M. (2020). Autoantibody-negative insulin-dependent diabetes mellitus after SARS-CoV-2 infection: a case report. Nat. Metab..

[16] Zeller I., Weiss A., Arnolds S., Schütte-Borkovec K., Arabi S., von dem Berge T., Casteels K., Hommel A., Kordonouri O., Larsson H.E., Lundgren M., Rochtus A., Snape M.D., Szypowka A., Vatish M., Winkler C., Bonifacio E., Ziegler A.-G. (2024). Infection episodes and islet autoantibodies in children at increased risk for type 1 diabetes before and during the COVID-19 pandemic. Infection.

[17] Gottesman B.L., Yu J., Tanaka C., Longhurst C.A., Kim J.J. (2022). Incidence of new-onset type 1 diabetes among US children during the COVID-19 global pandemic. JAMA Pediatr..

[18] Kamrath C., Rosenbauer J., Eckert A.J., Siedler K., Bartelt H., Klose D., Sindichakis M., Herrlinger S., Lahn V., Holl R.W. (2022). Incidence of type 1 diabetes in children and adolescents during the COVID-19 pandemic in Germany: results from the DPV registry. Diabetes Care.

[19] Weiss A., Donnachie E., Beyerlein A., Ziegler A.-G., Bonifacio E. (2023). Type 1 diabetes incidence and risk in children with a diagnosis of COVID-19. JAMA.

[20] Friedl N., Sporreiter M., Winkler C., Heublein A., Haupt F., Ziegler A.-G., Bonifacio E. (2024). Progression from presymptomatic to clinical type 1 diabetes after COVID-19 infection. JAMA.

[21] Ziegler A.-G., Achenbach P., Berner R., Casteels K., Danne T., Gündert M., Hasford J., Hoffmann V.S., Kordonouri O., Lange K., Elding Larsson H., Lundgren M., Snape M.D., Szypowska A., Todd J.A., Bonifacio E. (2019). Oral insulin therapy for primary prevention of type 1 diabetes in infants with high genetic risk: the GPPAD-POInT (global platform for the prevention of autoimmune diabetes primary oral insulin trial) study protocol. BMJ Open.

[22] Lugar M., Eugster A., Achenbach P., von dem Berge T., Berner R., Besser R.E.J., Casteels K., Elding Larsson H., Gemulla G., Kordonouri O., Lindner A., Lundgren M., Müller D., Oltarzewski M., Rochtus A., Scholz M., Szypowska A., Todd J.A., Ziegler A.-G., Bonifacio E. (2023). SARS-CoV-2 infection and development of islet autoimmunity in early childhood. JAMA.

[23] Krischer J.P., Lernmark Å., Hagopian W.A., Rewers M.J., McIndoe R., Toppari J., Ziegler A.-G., Akolkar B. (2023). SARS-CoV-2 - No increased islet autoimmunity or type 1 diabetes in teens. N. Engl. J. Med..

[24] Liu E., Lee H.-S., Aronsson C.A., Hagopian W.A., Koletzko S., Rewers M.J., Eisenbarth G.S., Bingley P.J., Bonifacio E., Simell V., Agardh D. (2014). Risk of pediatric celiac disease according to HLA haplotype and country. N. Engl. J. Med..

[25] Winkler C., Jolink M., Knopff A., Kwarteng N.-A., Achenbach P., Bonifacio E., Ziegler A.-G. (2019). Age, HLA, and sex define a marked risk of organ-specific autoimmunity in first-degree relatives of patients with type 1 diabetes. Diabetes Care.

[26] Cohen B.S., Lebwohl B. (2023). COVID-19 and celiac disease: a review. Therap. Adv. Gastroenterol..

[27] ElSayed N.A., Aleppo G., Aroda V.R., Bannuru R.R., Brown F.M., Bruemmer D., Collins B.S., Hilliard M.E., Isaacs D., Johnson E.L., Kahan S., Khunti K., Leon J., Lyons S.K., Perry M.L., Prahalad P., Pratley R.E., Seley J.J., Stanton R.C., Gabbay R.A. (2023). Classification and diagnosis of diabetes: standards of care in diabetes-2023. Diabetes Care.

[28] Ziegler A.G., Danne T., Dunger D.B., Berner R., Puff R., Kiess W., Agiostratidou G., Todd J.A., Bonifacio E. (2016). Primary prevention of beta-cell autoimmunity and type 1 diabetes - the Global Platform for the Prevention of Autoimmune Diabetes (GPPAD) perspectives. Mol. Metabol..

[29] Houben J., Janssens M., Winkler C., Besser R.E.J., Dzygalo K., Fehn A., Hommel A., Lange K., Elding Larsson H., Lundgren M., Roloff F., Snape M., Szypowska A., Weiss A., Zapardiel-Gonzalo J., Zubizarreta N., Ziegler A.-G., Casteels K. (2022). The emotional well-being of parents with children at genetic risk for type 1 diabetes before and during participation in the POInT-study, Pediatr. Diabetes.

