# Supplementary figures and images for: Anti-viral action against type 1 diabetes autoimmunity: The GPPAD-AVAnT1A study protocol
Source: Contemp Clin Trials Commun. 2025 Jan 20;44:101434. doi: 10.1016/j.conctc.2025.101434 (PMC11799962; doi:10.1016/j.conctc.2025.101434)

# Supplemental material

Appendix A_Schedule of procedure


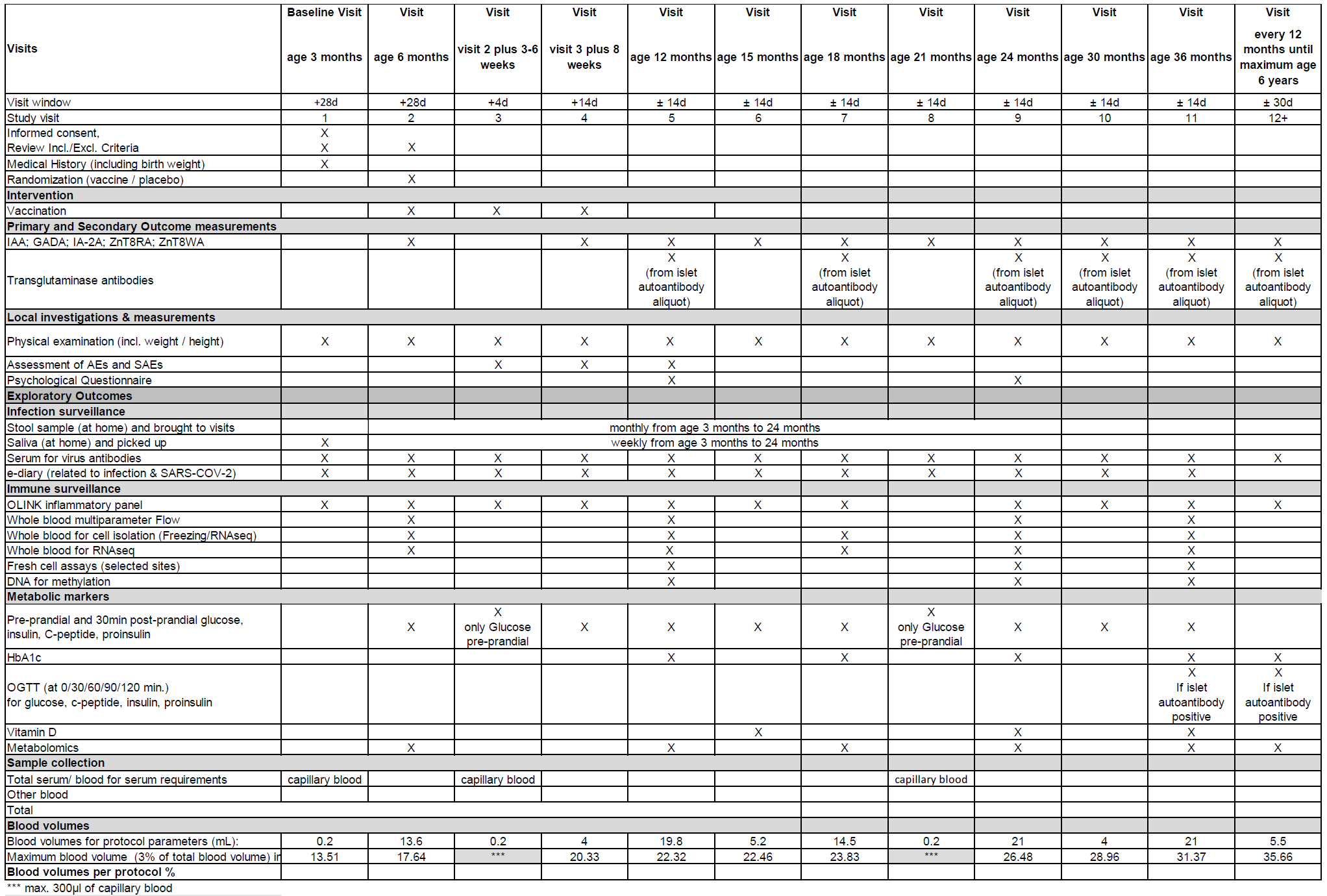

Supplement: Multimedia component 1 [file mmc1.docx]
